# Association of Preoperative Hemoglobin A1c with In-hospital Mortality Following Valvular Heart Surgery

**DOI:** 10.21470/1678-9741-2019-0320

**Published:** 2020

**Authors:** Mohammadreza Shoghli, Rajesh Jain, Mohamamdali Boroumand, Shayan Ziaee, Aras Rafiee, Leyla Pourgholi, Akbar Shafiee, Arash Jalali, Seyedeh Hamideh Mortazavi, Seyed Hossein Ahmadi Tafti

**Affiliations:** 1 Tehran Heart Center, Cardiovascular Diseases Research Institute, Tehran University of Medical Sciences, Tehran, Iran.; 2 Jain Hospital, Kanpur, India.; 3 Department of Biology, Central Tehran Branch, Islamic Azad University, Tehran, Iran.

**Keywords:** Diabetes Mellitus, Type 2, Glycated Hemoglobin A, Hospital Mortality, Cardiac Surgical Procedures, Elective Surgical Procedures, Blood Glucose, Hospitalization

## Abstract

**Objective:**

To determine the association between the preoperative level of hemoglobin A1c (HbA1c) and in-hospital mortality in patients who underwent valvular heart surgery in our center in a retrospective cohort.

**Methods:**

In this retrospective consecutive cohort study, patients with type 2 diabetes mellitus who were referred to our center for elective valvular surgery were enrolled and followed up. The endpoint of this study was in-hospital mortality. Based on the level of HbA1c, patients were dichotomized around a level of 7% into two groups: exposed patients with HbA1c ≥ 7% and unexposed patients with HbA1c < 7%. Then, the study variables were compared between the two groups.

**Results:**

Two hundred twenty-four diabetic patients who were candidates for valvular surgery were enrolled; 106 patients (47.3%) had HbA1c < 7%, and 118 patients (52.6%) had HbA1c ≥ 7%. The duration of diabetes was higher in patients with HbA1c ≥ 7% (*P*=0.007). Thirteen (5.8%) patients died during hospital admission, of which nine patients were in the high HbA1c group. There was no significant difference between the groups regarding in-hospital mortality (*P*=0.899). Both the unadjusted and adjusted logistic regression models showed that HbA1c was not a predictor for in-hospital mortality (*P*=0.227 and *P*=0.388, respectively)

**Conclusion:**

This study showed no association between preoperative HbA1c levels and in-hospital mortality in candidates for valvular heart surgery.

**Table:** 

Abbreviations, acronyms & symbols	
**ACE**	**= Angiotensin converting enzyme**
**ARB**	**= Angiotensin-receptor blockers**
**BMI**	**= Body mass index**
**CABG**	**= Coronary artery bypass grafting**
**CAD**	**= Coronary artery disease**
**CI**	**= Confidence interval**
**COPD**	**= Chronic obstructive pulmonary disease**
**FBS**	**= Fasting blood glucose**
**HbA1c**	**= Hemoglobin A1c**
**ICU**	**= Intensive care unit**
**LDL**	**= Low-density lipoproteins**
**NYHA**	**= New York Heart Association**
**OR**	**= Odds ratio**

## INTRODUCTION

The global incidence and prevalence of diabetes mellitus are dramatically increasing in recent years, and in 2011, almost 25% of the Iranian population had impaired fasting glucose or diabetes mellitus^[1,2]^. Control of blood glucose in patients who undergo any surgical operation is crucial as diabetes mellitus has been linked to the development of many adverse outcomes following open-heart surgeries, such as sepsis, wound infection, cerebrovascular accidents, postoperative atrial fibrillation, and mortality^[3-6]^. Therefore, a valid and reliable marker for the evaluation of diabetes control can help much to reduce these complications.

Hemoglobin A1c (HbA1c) has been introduced as a useful marker that can show the situation of glucose control in the past 8-12 weeks before the evaluation^[7]^. Hyperglycemia, as detected by elevated HbA1c, has been shown to be linked with major adverse events following cardiac surgery, ranging from sternal infection to death^[8]^. However, this effect was mostly observed in patients who underwent coronary artery bypass grafting (CABG), and data about the effect of hyperglycemia on valvular heart surgery is scarce.

Therefore, we aimed to determine the association between the level of HbA1c and in-hospital mortality in patients who underwent valvular heart surgery in our center in a retrospective cohort.

## METHODS

In this retrospective cohort study, consecutive patients with type 2 diabetes mellitus who were referred to our center for elective valvular surgery were enrolled and followed up. The inclusion criteria were: 1) being a candidate for valvular heart surgery alone; 2) having established type 2 diabetes; and 3) giving informed consent for taking part in the study. The exclusion criteria were: 1) emergency surgery; 2) history of cardiac surgery; 3) simultaneous CABG; 4) history of chronic inflammatory disease, malignancy, or hepatic failure; and 5) history of hemolytic anemia, hemoglobinopathy, or recent massive bleeding. Based on our hospital policy, all of the patients signed a written informed consent at the time of their admission, allowing anonymous use of their clinical data for research purposes. Our study protocol was approved by the Research Board and committee of Medical Ethics of the Tehran Heart Center. This study was performed in accordance with the latest version of the declaration of Helsinki.

All of the demographic and clinical data of the patients were retrieved from the cardiac surgery database of the Tehran Heart Center^[9]^. At the time of admission, the height and weight of the patients were measured, and body mass index was calculated for every patient. A thorough history of habits, cardiac risk factors, and previous medical conditions was also obtained and recorded. Transthoracic echocardiography was performed before the operation, and the patients’ ejection fraction was used for this study.

On the day of the surgery, a fasting venous blood sample was obtained from every patient for biochemistry tests, especially fasting blood glucose (FBS) and HbA1c. These measurements were performed by turbidimetric inhibition immunoassay using Cobas Integra 400 plus.

Valvular surgeries were performed under the highest standards in the operation room of the Tehran Heart Center. After anesthesia and prep and drape, a median sternotomy was performed. The aorta, superior vena cava, and inferior vena cava were emulated. Based on the valvular problem, the patients underwent valvular replacement or repair in one or more valves.

Following surgery, all patients underwent cardiac monitoring in the intensive care unit (ICU) and the post-ICU ward for the development of any complication, particularly mortality. Based on the level of HbA1c, patients were dichotomized around a level of 7% into two groups: exposed patients with HbA1c ≥ 7% and unexposed patients with HbA1c < 7%. Then, the study variables, including the frequency of mortality, were compared between the two groups. The endpoint of this study was in-hospital mortality.

### Statistical Analysis

Mean ± standard deviation or median with quartiles and frequency (percentage) were used to describe continuous and categorical variables, respectively. Continuous variables were compared between HbA1c groups using Student’s t-test or Mann-Whitney U test. Categorical variables were compared between the mentioned groups using the chi-square or Fisher’s exact test. The association of Hba1c levels and mortality was adjusted on detected possible confounders in this study using a logistic regression model, and it was expressed as an odds ratio (OR) with a 95% confidence interval (CI). P-values ≤ 0.05 were considered statistically significant. The IBM SPSS Statistics software, version 21.0 (Microsoft, United States of America), was used for statistical analyses.

## RESULTS

In this study, 224 diabetic patients who were candidates for valvular surgery were enrolled. Mitral and tricuspid valves were the most common valves that were operated, and in 112 patients, more than one valve was operated (Table 1). One hundred six patients (47.3%) had HbA1c < 7% and 118 patients (52.6%) had HbA1c ≥ 7%. There was no significant difference between the groups in demographic and clinical characteristics, including cardiovascular risk factors (Table 2). However, the levels of triglyceride and total cholesterol were significantly higher in patients with high HbA1c (*P*<0.001 and *P*=0.005, respectively). The groups were not statistically different regarding other laboratory indices, ventilation time, and ICU admission.

**Table 1:** Frequency of the types of valvular surgery in the study population (n=224).

Valve/type of surgery	Frequency (percentage)
Aortic valve	89 (39.7)
Replacement	82 (36.6)
Reconstruction	3 (1.3)
Repair	4 (1.8)
Mitral valve	147 (65.6)
Annuloplasty	4 (1.8)
Repair	135 (60.3)
Reconstruction with annuloplasty	3 (1.3)
Reconstruction without annuloplasty	5 (2.2)
Pulmonary valve	7 (3.1)
Repair	5 (2.2)
Reconstruction	2 (0.9)
Tricuspid valve	93 (41.5)
Annuloplasty	42 (18.8)
Replacement	24 (10.7)
Reconstruction with annuloplasty	6 (2.7)
Reconstruction without annuloplasty	21 (9.4)

**Table 2:** Comparison of the baseline study variables between the study groups.

Characteristic*	Controlled diabetes (n=106)	Uncontrolled diabetes (n=118)	*P*-value†
Age, years	60.5±8.7	58.0±8.9	0.034
Male gender, n (%)	33 (31.1)	38 (32.2)	0.863
BMI, kg/m^2^	27.0±4.7	27.3±4.8	0.642
Hypertension, n (%)	55 (51.9)	65 (55.1)	0.632
Dyslipidemia, n (%)	56 (52.8)	72 (61.0)	0.216
Family history of CAD, n (%)	36 (34.0)	32 (27.1)	0.266
Smoking, n (%)	5 (4.7)	5 (4.2)	0.999
COPD, n (%)	9 (8.5)	5 (4.2)	0.189
Duration of diabetes, years	3.0 [1.0, 5.0]	5.0 [3.0, 10.0]	0.007
Diabetes treatment, n (%)			0.21
None	13 (14.1)	7 (6.5)	
Diet	5 (5.4)	2 (1.9)	
Oral agent	65 (70.7)	79 (73.1)	
Insulin	9 (9.8)	13 (12.0)	
Combination therapy	0 (0)	7 (6.5)	
Arrhythmia, n (%)	84 (79.2)	97 (82.9)	0.485
Atrial fibrillation, n (%)	41 (38.7)	33 (28.0)	0.089
Beta blocker, n (%)	32 (30.2)	33 (28.0)	0.714
Calcium channel blocker, n (%)	94 (88.7)	94 (79.7)	0.067
Nitrates, n (%)	87 (82.1)	91 (77.1)	0.359
Diuretics, n (%)	40 (37.7)	44 (37.3)	0.945
ACE inhibitor/ARB, n (%)	82 (77.4)	93 (78.8)	0.793
Aspirin, n (%)	61 (57.5)	68 (57.6)	0.990
Antiplatelet, n (%)	105 (99.1)	116 (98.3)	0.999
Anticoagulant, n (%)	46 (43.4)	43 (36.4)	0.288
Inotropes, n (%)	105 (99.1)	116 (98.3)	0.999
Fasting blood sugar, mg/dl	105.0 [86.0, 139.0]	187.0 [157.0, 224]	<0.001
Blood urea nitrogen, mg/dl	41.0 [34.0, 55.0]	42.0 [34.9, 56.0]	0.965
Creatinine, mg/dl	0.90 [0.74, 1.10]	0.82 [0.71, 1.10]	0.432
Triglyceride, mg/dl	109.0 [82.0, 157]	146.0 [108.0, 188.0]	<0.001
Total cholesterol, mg/dl	142.5 [118.0, 171.0]	162.0 [132.0, 188.0]	0.005
LDL, mg/dl	83.0 [67.0, 113.0]	103.5 [74.0, 122.0]	0.1
HDL, mg/dl	40±14.0	37.5±9.6	0.13
Hemoglobin, mg/dl	12.8±1.6	13.1±1.9	0.134
Na, mg/dl	142.7±3.7	142.0±4.8	0.582
K, mg/dl	4.4±0.5	4.4±0.5	0.565
Ejection fraction, %	47.6±9.5	48.4±9.2	0.497
Hemoglobin A1c, mg/dl	6.27±0.48	8.86±1.39	<0.001
NYHA classification, n (%)			0.232
I	14 (14.3)	14 (14.3)	
II	48 (49.0)	56 (57.1)	
III	28 (28.6)	26 (26.5)	
IV	8 (8.2)	2 (2.0)	
Pump time, min	90.0 [70.0, 130.5]	93.0 [67.0, 120.0]	0.584
Cross-clamp time, min	58.0 [42.0, 82.0]	56.0 [43.5, 74.5]	0.961
Ventilation duration, hours	11.2 [8.5, 15.0]	12.0 [8.5, 15.5]	0.636
ICU stay, hours	51.7 [25.0, 116.5]	47.7 [25.0, 96.0]	0.735

* *P*-value < 0.05 was considered as statistically significant

† Variables with normal distribution are shown as mean±standard deviation, and distorted distributed variables are shown as median [interquartile range]

ACE=angiotensin converting enzyme; ARB=angiotensin-receptor blockers; BMI=body mass index; CAD=coronary artery disease; COPD=chronic obstructive pulmonary disease; HDL=high-density lipoproteins; ICU=intensive care unit; LDL=low-density lipoproteins; NYHA=New York Heart Association

The median duration of hospital stay was 13.4 days, and 13 (5.8%) patients died during hospital admission, of which nine patients were in the high HbA1c group. Five patients died due to heart failure, four patients had sternal wound infection, one patient had gastrointestinal bleeding, one patient developed acute respiratory distress syndrome, one patient had sepsis, and one patient developed cerebrovascular accident during hospitalization. However, there was no significant difference between the groups regarding in-hospital mortality (*P*=0.899). The unadjusted logistic regression model showed that HbA1c was not a predictor for in-hospital mortality (OR: 2.1, CI: 0.62-7.05; *P*=0.227) (Table 3). After adjustment for age, duration of diabetes, triglyceride, and low-density lipoprotein, HbA1c did not present any association with in-hospital mortality (OR: 2.07, CI: 0.39-10.91; *P*=0.388) (Table 3).

**Table 3:** The unadjusted and adjusted regression model for the predictive effect of hemoglobin A1c on in-hospital mortality.

Characteristic	Odds ratio	95% confidence interval	*P*-value*
Unadjusted			
In-hospital mortality	2.1	0.62-7.05	0.227
Adjusted†			
In-hospital mortality	2.07	0.39-10.91	0.388

* *P*-value < 0.05 was considered as statistically significant

† Adjusted for age, diabetes duration, low-density lipoprotein, and triglyceride level

## DISCUSSION

This study aimed to find the association between HbA1c levels and mortality following valvular heart surgery. However, we did not observe this association.

High blood glucose at the time of surgery has been reported as a risk factor for developing adverse events, such as longer ICU stay, longer ventilation time, and atrial fibrillation following open-heart surgery^[10]^. It has been shown that tight blood glucose control at the time of heart surgery has been accompanied by lower adverse outcomes following CABG^[11]^. The only study that was solely performed on patients with valvular heart surgery also showed no association between the level of HbA1c and major adverse cardiac events (including mortality)^[12]^. Additionally, although patients with HbA1c > 6.5 had more considerable glycemic variability in the mentioned study, this did not lead to a higher incidence of hyperglycemia or hypoglycemic episodes. Wang et al.^[13]^ showed that HbA1c is the only variable of diabetes that can independently predict long-term mortality following CABG. This supports the concept that rapid glucose adjustment right before the surgery is less beneficial than long-term glycemic control. Based on Halkos et al.^[14]^ study, HbA1c > 8.6% can involve a four-times risk of perioperative mortality in CABG patients. Another study has shown that not only can the elevated HbA1c predict poorer outcomes following cardiac surgery, but also it is more observed in patients in low socioeconomic patterns ^[15]^. Hudson et al.^[16]^ showed in their study on non-diabetic patients who underwent elective cardiac surgery that elevated HbA1c is directly associated with higher short-term mortality. Some other similar studies also suggested the association between elevated HbA1c and mortality following cardiac surgery^[17-19]^.

It should be noted that most of the studies on the association of glycemic control with the outcomes of cardiac surgery have been performed on candidates for CABG, and this limits our ability to compare our findings with previously published data. As coronary artery disease is caused by atherosclerosis, a direct result of diabetes, most of these studies showed an independent association between elevated HbA1c and a higher rate of mortality and adverse outcomes. As atherosclerosis is not a common etiology for the valvular disease^[20]^, this can rationalize the findings of our study.

### Study Limitations

The retrospective nature of our study limited our ability to find out more data on the in-hospital events and post-discharge survival of the patients. On the other hand, our center is a tertiary heart center, and it is probable that most referred patients were in a worse condition than the general population. Moreover, we did not have the data on the diabetes treatment and duration of diabetes in our study population to see whether the type of treatment and duration of the disease were related to in-hospital outcome and mortality. Finally, we only studied the in-hospital mortality of the patients, and it is presumed that by having longer follow-up results, we could find different results.

## CONCLUSION

Overall, this study showed no association between preoperative HbA1c levels and in-hospital mortality in candidates for valvular heart surgery. Future studies with a larger sample size should study the effect of HbA1c and glycemic control on the long-term survival of candidates for valvular heart surgery.

**Table:** 

Authors' roles & responsibilities	
MS	Substantial contributions to the conception or design of the work; or the acquisition, analysis, or interpretation of data for the work; drafting the work or revising it critically for important intellectual content; final approval of the version to be published
RJ	Substantial contributions to the conception or design of the work; or the acquisition, analysis, or interpretation of data for the work; drafting the work or revising it critically for important intellectual content; final approval of the version to be published
MB	Substantial contributions to the conception or design of the work; or the acquisition, analysis, or interpretation of data for the work; drafting the work or revising it critically for important intellectual content; final approval of the version to be published
SZ	Substantial contributions to the conception or design of the work; or the acquisition, analysis, or interpretation of data for the work; drafting the work or revising it critically for important intellectual content; final approval of the version to be published
AR	Substantial contributions to the conception or design of the work; or the acquisition, analysis, or interpretation of data for the work; drafting the work or revising it critically for important intellectual content; final approval of the version to be published
LP	Substantial contributions to the conception or design of the work; or the acquisition, analysis, or interpretation of data for the work; drafting the work or revising it critically for important intellectual content; final approval of the version to be published
AS	Substantial contributions to the conception or design of the work; or the acquisition, analysis, or interpretation of data for the work; drafting the work or revising it critically for important intellectual content; final approval of the version to be published
AJ	Substantial contributions to the conception or design of the work; or the acquisition, analysis, or interpretation of data for the work; drafting the work or revising it critically for important intellectual content; final approval of the version to be published
SHM	Substantial contributions to the conception or design of the work; or the acquisition, analysis, or interpretation of data for the work; drafting the work or revising it critically for important intellectual content; final approval of the version to be published
SHAT	Substantial contributions to the conception or design of the work; or the acquisition, analysis, or interpretation of data for the work; drafting the work or revising it critically for important intellectual content; final approval of the version to be published
